# Unlocking Pandora's box: personalising cancer cell death in non-small cell lung cancer

**DOI:** 10.1186/1878-5085-3-6

**Published:** 2012-06-18

**Authors:** Dean A Fennell, Charles Swanton

**Affiliations:** 1 University of Leicester & Leicester University Hospitals, Hodgkin Building, Lancaster Road, PO Box 138, Leicester, LE1 9HN, UK; 2 Cancer Research UK London Research Institute, Translational Cancer Therapeutics Laboratory, 44 Lincoln's Inn Fields, London, WC2A 3LY, UK; 3 UCL Cancer Institute, Paul O'Gorman Building, Huntley St, London, WC1E, UK

**Keywords:** Personalised medicine, Lung cancer, Somatic mutations, Apoptosis, Mitochondria, Targeted therapy

## Abstract

Evasion of apoptosis is a hallmark of tumorigenesis and a recognised cause of multidrug resistance. Over the last decade, insights into how apoptosis might be exploited in non-small cell lung cancer (NSCLC) and how cancer therapeutics might be used to engage apoptotic signalling in a personalised manner have changed markedly. We are now in the wake of a paradigm shift in stratified therapeutic approaches related to NSCLC. At the heart of this shift in thinking is the emerging knowledge that even the most drug-resistant cancers exhibit a functional death pathway and, critically, that this pathway can be efficiently engaged, leading to clinical benefit. This review will summarise current knowledge of mitochondrial apoptotic pathway dysfunction in NSCLC and how the next generation of targeted therapeutics might be used to exploit deficiencies in apoptotic signalling in a personalised manner to improve clinical outcome and predict therapeutic benefit.

## Review

The mitochondria collectively represent a cellular *Pandora's box*. The key to effective treatment of non-small cell lung cancer (NSCLC) should be the identification of personalised therapeutic approaches capable of selectively engaging mitochondrial cell death. An approach that holds the greatest promise for achieving this may lie in the use of genetics to identify ‘keys to unlock’ Pandora's box in NSCLC.

### Pandora's box and mitochondrial apoptosis

The organelles termed mitochondria constitute a major component of the canonical death pathway which initiates apoptosis. During life, these organelles play a critical role in maintaining bioenergetic homeostasis, predominantly through the synthesis of adenosine trisphosphate. However, following a fatal cellular insult, mitochondria commit irreversibility to ensuring the death of the cell. To achieve this, mitochondria release into the cytoplasm death-signalling proteins, which are normally harboured, safe out of reach, within the intermembrane space or cristae. This occurs as a result of mitochondrial outer membrane permeabilisation or MOMP. Upon release, these factors which include cytochrome C [1,2], OMI, smac [3], and apoptosis-inducing factor [4,5] lead to the activation of cellular demolition machinery comprising zymogens termed caspases, which systematically cause the noninflammatory elimination of the cell via apoptosis and immune cell engulfment [6]. MOMP occurs as a result of the activation of proapoptotic BCL2 family proteins.

The multidomain family members BAX and BAK exhibit genetic redundancy and undergo homo- and hetero-oligomerisation [7,8] to promote pore formation in the outer mitochondrial membrane — essentially the key to unlocking Pandora's box [9]. To trigger MOMP, BAX/BAK must first be activated. This requires a subset of proapoptotic BCL2 family proteins which harbour a single BCL-2 homology domain 3 (BH3) [10-13]. This alpha helical amphipathic domain is sufficient to activate BAX and BAK and may do so by two mechanisms. The BH3-only proteins BIM, BID and PUMA directly activate BAX/BAK, leading to their oligomerisation and MOMP [14,15]. BIM is activated by post-translational modification through phosphorylation, causing its stabilisation [16-19]. In contrast, BID is a substrate for caspase 8 cleavage induced by death receptor ligation [20], whereas PUMA is transcriptionally activated [21]. A second group of five BH3-only proteins (BAD, NOXA, BNIP3, BFK and BMF) activates BAX and BAK by disrupting their interaction with the prosurvival members of the BCL2 family, which comprise BCL2, BCLXL, MCL1, BCLW and BCLB [22-25]. The apparent redundancy in BH3-only proteins may be explained by their role as functionally distinct cell damage sensors.

### Do prosurvival BCL2 proteins ‘lock’ Pandora's box?

Prosurvival BCL2 family proteins have long been considered as critical suppressors of apoptosis in cancer. This is supported by their high rate of gene amplification. For example, MCL1 encoded at the 1q21.2 locus frequently exhibits copy number gain in cancer, followed by BCLX (BCLL2) [26]. The basis for this may be as a countermeasure to circumvent the proapoptotic activity of critical oncogenes during transformation. For example, c-myc-driven transformation has been shown to require obligatory upregulation of BCL2 to overcome CD95-mediated apoptosis [27,28]. More recently, it has been shown that H-RAS-mediated transformation leads to non-apoptotic cell death through induction of autophagy with upregulation of the BH3-only protein NOXA and disruption of the MCL-1-beclin-1 complex [29]. Overexpression of prosurvival BCL2 family members may therefore reflect a required defence against constitutively generated ‘death signals’, which would otherwise induce apoptosis. Growing evidence suggests that constitutive activation of BH3 death signals probably induced by ‘insufficient oncogenes’ results in addiction to prosurvival BCL2 family proteins. It has been proposed that prosurvival BCL2 members, by forming constitutive complexes with activated BH3-only proteins, are primed for death [30]. Accordingly, BH3 domain peptides, capable of competitively inhibiting heterodimerisation of prosurvival BCL2 family members, can induce apoptosis by freeing BAX and BAK to oligomerise [31]. These BH3-only domains exhibit restricted specificity for prosurvival BCL2 members. For example, NOXA interacts only with MCL1 and A1, whereas BAD interacts with BCL2, BCLXL and BCLW. In contrast, BIM and PUMA are promiscuous in their prosurvival BCL2 protein interactions [32,33].

BH3 peptidomimetics are a new class of drug which were developed to mimic the interaction of BH3 domains. The prototype inhibitors navitoclax (ABT-263) [34] and ABT-737 were identified as BAD BH3 peptidomimetics using NMR-based screening, with high affinity for BCL2, BCLX and BCLW [35]. ABT-737 is a potent apoptosis sensitiser in preclinical models and can induce the regression of xenografts [36], which are primed for death such as small cell lung cancer. ABT-263 exhibits some evidence of efficacy in the clinical setting [37]; however, in common with other targeted agents, its efficacy may be limited in unselected populations. Identifying which cancers will respond to ABT-263 may be feasible. Because of the prosurvival BCL2 family restriction associated with ABT-263/737, there is robust preclinical evidence showing that MCL1 expression is a major resistance factor [38-42]. However, even in the presence of MCL1, ABT737 can exhibit preclinical activity in some cancers such as CLL, possibly due to constitutive occupancy due to priming for death. This possibility is reflected in gene expression analysis, which has shown a correlation between sensitivity and the expression of NOXA [43]. In preclinical models of lung cancer and mesothelioma, cell lines grown in three dimensions acquire apoptosis resistance [44] due to an altered expression of prosurvival BCL2 family members conferring sensitivity to ABT737, and this is associated with priming for death [45,46].

### Growth factor suppression of the core apoptosis machinery

The mitochondrial apoptosis pathway is directly suppressed by survival signalling. BIM expression is regulated by phosphorylation in response to growth factors [16-19,47-50]. This occurs in a mitogen-activated protein kinase kinase-extracellular signal-regulated protein kinase 1/2 (MAPK-ERK)-dependent manner, leading to its polyubiquitination and destabilisation through proteasomal degradation [16]. The dissociating BH3-only protein BAD is regulated by serine phosphorylation, which causes its inactivation through sequestration to 14-3-3 [51].

The prosurvival BCL2 protein MCL-1 is also regulated by survival signals. The phosphatidylinositol 3-kinase/protein kinase B (PI3K/AKT) pathway, which signals downstream of receptor tyrosine kinases such as the epidermal growth factor receptor, stabilises MCL-1 via AKT signalling. Glycogen synthase kinase 3 (GSK-3) is a substrate for and is inhibited by AKT. Upon growth factor withdrawal, GSK-3 is de-repressed and phosphorylates MCL-1 at S159. This phosphorylation leads to ubiquinylation and degradation of MCL-1 via the proteasome. Collectively therefore, survival and proliferation are driven in parallel with suppression of apoptosis. Recently, it has emerged that dominant survival pathways exist in subsets of NSCLC, which, if targeted, can unleash BH3-only proteins and mediate effective apoptosis both at the bench and at the bedside.

### Dominant oncogenes as Achilles' heels for unleashing BH3-only proteins

Although there is overwhelming evidence now to implicate genomic instability and temporal acquisition of complex somatic gene alterations as causal factors during carcinogenesis, the identification of critical oncogenic drivers has had major implications for the development of therapeutics in NSCLC. The paradigm in this solid tumour mirrors the discovery and targeting of the BCR-ABL fusion protein, the dominant oncogenic driver in chronic myelogenous leukaemia [52-55]. Prior to 2004, a therapeutic plateau had been reached in the management of NSCLC. Platinum-based therapy was considered the gold standard with no identifiable superior regimen, associated modest response rates and impact on overall survival [56] even with the addition of novel agents [57,58]. Clinical trials then focused on what was considered a homogeneous NSCLC population. Although this approach led to the approval of the epidermal growth factor receptor tyrosine kinase inhibitor (EGFR TKI) erlotinib in second and third line NSCLC [59,60], this was not reflected in the negative, front-line pivotal phase III trials combining EGFR TKIs gefitinib and irressa with chemotherapy in unselected populations [61-64]. The seminal discovery that a subset of patients harbouring somatic mutations of the EGFR exhibits dramatic responses to the orally bioavailable receptor tyrosine kinase inhibitor gefitinib or erlotinib [65-67] spearheaded the shift in thinking about how to target NSCLC more effectively. The superior efficacy of oral TKI therapy for treating mutant EGFR (EGFR^MUT^)-positive NSCLC was borne out in pivotal randomised, controlled clinical trials [68,69].

Somatic mutations of EGFR, of which the most common are deletions of exon 19 and nucleotide substitions in exon 21 [70], have been shown to confer an increase in anti-apoptotic signalling capacity [71] and confer a cell survival advantage driving Darwinian selection. Mutation of EGFR causes an increase in enzyme activity and suppression of autoinhibitory function [72] and confers resistance to conventional cytotoxic drugs [71]. Inhibition leads to dramatic activation of apoptosis via the mitochondrial pathway [73] involving activation of BIM in common with the apoptosis that results following the inhibition of bcr-abl kinase in CML [55,74]; this is consistent with a common link between driver oncogene addiction, survival and suppression of critical death signals.

**Figure 1:**
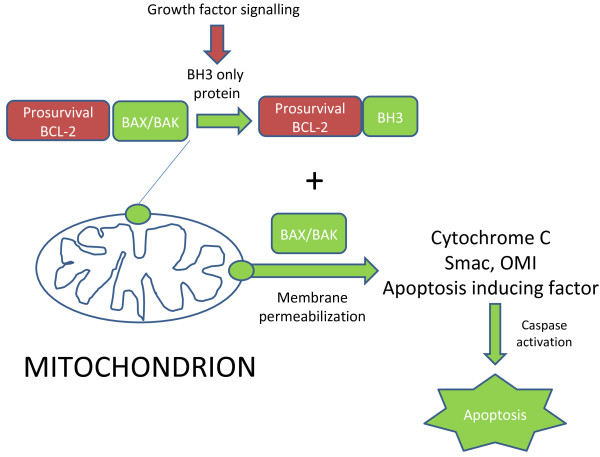
**Mitochondrial apoptosis pathway.** Mitochondria are effectors for cell death following inhibition of growth factor-addicted survival pathways linked to somatic oncogene mutations (eg., EGFR). BH3-only proteins are unleashed and lead to the activation of the mitochondrial apoptosis pathway. Proapoptotic signalling is indicated in green, anti-apoptotic in red.

Upon inhibition of EGFR^MUT^, there is a dramatic increase in the level of BIM through transcriptional and post-translational mechanisms (Figure 1). Activation of BIM requires the MAPK-ERK signalling pathway, but not JNK nor PI3K/AKT signalling. Silencing this BH3-only protein alone effectively rescues from EGFR^MUT^ inhibition in vitro and in vivo [75-78]. ABT-737 confers sensitivity to erlotinib in EGFR^MUT^ cells [75,77], implicating a potential for BH3 peptidomimetics as enhancers of EGFR TKIs efficacy in common with BIM-mediated apoptosis in bcr-abl-inhibited CML [55]. However, one caveat to this approach could be the rapidly emerging resistance and failure to activate critical death signals.

### BIM mediates apoptosis following ALK inhibitor in EML4-ALK-rearranged NSCLCs

Within 3 years of the initial discovery of EGFR^MUT^ addiction in NSCLC, a small proportion of NSCLCs (around 4 % to 6 %) was reported to harbour a small inversion within chromosome 2p, resulting in the fusion of the echinoderm microtubule-associated protein-like 4 (EML4) gene and the anaplastic lymphoma kinase (ALK) gene. As with EGFR^MUT^ NSCLC, these NSCLCs are restricted to a specific histological subset of NSCLCs, namely non-squamous tumours. Consistent with EML4-ALK being a driver mutation, NSCLCs harbouring this gene rearrangement are mutually exclusive of EGFR or KRAS mutations. The mechanism underlying induction of apoptosis following inhibition of ALK involves the upregulation of BIM and downregulation of the inhibitor of apoptosis protein survivin [79]. EML4-ALK drives the ERK pathway, and this is involved in the suppression of BIM, in contrast to surviving which is regulated by the STAT pathway in response to EML4-ALK. Consequently, targeting EML4-ALK appears to induce apoptosis, in common with EGFR inhibition, through activation of intrinsic mitochondrial pathway. Based on the dramatic responses seen in the clinical setting [80], the ALK inhibitor crizotinib (PF-02341066) received approval in record time [80].

### Resistance to targeted therapies through emergent secondary mutations blocks apoptosis

In the case of mutant EGFR, clinical efficacy is frequently limited by secondary mutation. The most commonly described mutation T790M [81,82], which can also be transmitted in the germline, is also a susceptibility allele [83,84]. T790M is present as a minor clone in NSCLC [85,86] and may be selected for during therapy [87]. This mutation has been shown to prevent the activation of BIM in response to gefitinib but can be overcome by an irreversible inhibitor of EGFR [76]. Other critical resistance mechanisms have been identified to be linked to the inhibition of EGFR^MUT^ and include MET amplification [88-91], PTEN loss [92], HER2 kinase domain [93] or PIK3CA, and transformation to small cell lung cancer [87]. Selection pressure mediated by dramatic apoptosis induction drives the emergence of resistant clones capable of abrograting apoptosis signalling. Strategies to overcome this problem include structure-based modelling and discovery of *bona fide* T790M EGFR-targeting inhibitors [94]. Bypassing the EGFR altogether and target downstream pathways is another possibility. Survival signals from EGFR diverge through the PI3K/AKT and MAPK/ERK pathways. In EGFR^MUT^ cell lines, inhibition of PI3K/AKT pathway leads to selective downregulation of MCL-1. In contrast, inhibition of the MAPK/ERK pathway leads to BIM upregulation. Combining PI3K and MEK inhibition may therefore present a strategy for overcoming resistance [95], as might targeting dependence on NF kappa B, which has been identified by RNAi screening in the context of EGFR^MUT^[96].

In common with resistance mechanisms identified for EGFR (and indeed bcr-abl), ALK-rearranged cancers have been reported to develop secondary mutations [97,98]. Rearranged ALK is relatively unstable and is dependent upon heatshock protein 90 to prevent its spontaneous ubiquitination and targeting to the proteasome for degradation. Inhibition of the chaperone, heatshock protein 90 (HSP90), leads to a downregulation of EML4 ALK and inhibition of signalling even in the presence of secondary mutations, suggesting that this might be a potential strategy for overcoming resistance [99-101]. As with EGFR, structure-based modelling has potential to identify small-molecule inhibitors capable of overcoming resistance due to gatekeeper mutation of ALK [102].

### Personalising NSCLC therapy: towards identifying the full complement of oncogenic drivers in NSCLC

Beyond EGFR and ALK rearrangement, several additional somatic gene alterations linked to oncogenic drivers have been and are continued to be identified across the genomic landscape of NSCLC. As with the prototypical somatic mutations in EGFR and ALK, many of these may provide real opportunities for achieving similarly dramatic therapeutic outcomes. Sequencing efforts have identified mutations of KRAS, B-RAF, Her2/erbb2, PIKC3A, LKB1 and MET amplification [103-105]. These commonly occurring mutations can be screened in a clinical-practice-based setting to enable personalised therapy to be most effectively delivered [106]. For many or all of these oncogenic drivers, inhibition of the dominant signalling pathway may trigger BIM-dependent apoptosis, as shown for mutant B-RAF [107-109], Her2 [110] and MET amplification. KRAS is frequently mutated in around a quarter of NSCLCs, and its activation causes lung cancer in mice [111]. It has been widely recognised as a hard target to drug [112]. However, this situation is changing. For example, C-RAF, but not B-RAF, has been identified through gene ablation studies, and TBK1 through RNAi screening, to be essential for KRAS-dependent NSCLC. Stability of mutant KRAS may depend on HSP90 [113-115], implicating this chaperone as a potential molecular target. Finally, novel specific inhibitors of KRAS have been identified.

There have been rapid advances in genome sequencing technology, which now provide an opportunity to systematically interrogate somatic gene alterations in NSCLC [116,117]. The International Cancer Genome Consortium has been developed to do this in NSCLC and 49 other cancers [118]. With this technological development, it is likely that there will be the ability to comprehensively annotate the full complement of somatic gene alterations involved in driving NSCLC. The translational implications for personalised therapeutics and predictive biomarker development are clear based on the previous successes: that early identification of these oncogenic drivers will reflect vulnerabilities that will allow stratification of patients to receive the appropriate and effective apoptosis-inducing therapy.

## Conclusions

The induction of apoptosis has long been cited as a key objective for achieving drug-induced tumour suppression; however, evasion of this process has equally been known to limit the efficacy of conventional treatments. Now, in this era of genomics, it is clear that personalising drug therapy to most effectively target addiction to growth factor signalling pathways, by virtue of somatic mutations, provides a new exciting therapeutic opportunity, at least for initial disease control. However, combating resistance even in this era will present a major new challenge.

## Competing interests

The authors declare that they have no competing interests.

## Authors’ contribution

DF and CW jointly co-authored, read and approved the final manuscript.
